# A Hospital Inquisition

**Published:** 1890-04-26

**Authors:** 


### The Hospital

A Weekly Journal of

**Science, Medicine, Nursing, and Philanthropy.**

*For Hospitals, Asylums, Medical Practitioners, Students, Nurses, Families, Parishes, Congregations, and the Charitable Public.*

Vol. VIII.—No. 187.] [April 26, 1890.

### A Hospital Inquisition.

A period of inquisition is approaching for hospitals, and the modern Duke of Alva is the Charity Organization Society. Hospitals must, like the towns of the Netherlands, be up and doing, or the new "Blood Council" will have things all its own way. We do not need to inform managers and officials that the House of Lords under Lord Sandhurst's inspiration has consented to appoint a Committee to inquire into the constitution and working of all kinds of medical charities in the Metropolis. Although Lord Sandhurst is the ostensible leader in the movement it is an open secret that the Charity Organization Society is the vital breath and energy.

We cannot pretend to feel either surprise or regret at the impending investigation. For years past the air of medical London has been filled with the cries and shouts of battle. The hospital system, like some old feudal castle, has been assailed by Knights in black armour, Wilfreds of Ivanhoe, Locksleys, Gurths, and Witless Wambas. The mere noise of the raging fight has compelled public attention both to assailants and defenders. The castle has not yet fallen, nor is it likely to fall. But the present seems a not inappropriate time for a cessation of hostilities; and the Lords' Committee may very properly hold an inquiry as to the causes of the strife.

It will be more than a pity, it will be a blunder to restrict the inquiry to the hospitals of London as is proposed to be done. There is neither logic, expediency, nor practical sense in such a restriction. Whatever necessity exists in the Metropolis for the investigation exists in as great if not a greater measure throughout the county. Lord Sandhurst probably knows what a rick-yard is: no doubt he has, himself, one or more. If a band of incendiaries were to simultaneously set fire to all the ricks in any given yard, would Lord Sandhurst consider his farm bailiff a philosopher or a fool if he quenched one of the blazing piles and left all the others to be burnt to the ground and to spread conflagration on all hands? The whole country is a rick-yard of hospitals; and incendiaries, in the shape of dissatisfied general practitioners, are carrying blazing torches in all directions. If it is desirable to restore contentment and good order in London, on what conceivable ground can it be considered expedient to keep the fires of discontent smouldering or blazing in the country? It would be much better to have no inquiry at all than a partial inquiry. Government will reduce the Commission to the dimensions of a local vestry if London alone is to be dealt with, and the country left to its fate. Already the injured practitioners of Liverpool and other important towns are protesting with the utmost power of their lungs and pens against the incomprehensible limitation that is proposed.

All watchful defenders of medical charities should now look to the strengthening of their defences. Nobody pretends that there are not weak places both in the constitution and management of many hospitals in this country. It is the very madness of fatuity for the defenders to shut their eyes to breaches in their walls or to unworthiness in the garrison. The facts, with all that springs from them, must be looked squarely in the face. Surveying the hospital system and its outworks with critical eye, no man, be he assailant or be he defender, can fail to notice one part of that system which conspicuously invites attack. That weak place is the out-patient department. It has been so injured from without and from within that it has now come to be hardly defensible. From without patients have charged themselves upon its resources in countless numbers, who have had justification neither in their poverty nor in the nature of their illness. It cannot be denied that a very considerable proportion of hospital out-patients could well afford to pay ordinary doctors for their treatment, and a still larger proportion could do very well without any treatment at all. From within this department is almost as much injured as from without. The kind of practice that is often carried on there is a disgrace to the medical profession. The number of cases that a "physician"—save the mark!—will see, examine, and treat in an hour is incredible, and is absolutely destructive of professional honesty. The plain truth is that some out-patient physicians do not care one straw for their out-patient work except in so far as it is a stepping-stone to a permanent hospital appointment. It is certain, notwithstanding what some eminent and well-meaning physicians may have said to the contrary, that the abuse of the out-patient department at many hospitals is, to use the language of a famous orator, "gross as a mountain, open, palpable"; it is "a mockery, a delusion, and a snare." If this department did not already exist, there is not one of its defenders who would of free choice create it. All who look at it with honest and unprejudiced eyes insist that it must be "mended or ended."

The Committee will find it desirable to enter upon a searching investigation of the question of hospital book-keeping. No one is particularly to be blamed if the methods in vogue are almost as numerous and varied as the hospitals themselves. It must be remembered that the hospital system has sprung up, so to speak, *per naturam;* and it ought not, therefore, to surprise anybody if, like a natural forest, it shows a good deal of rough undergrowth, and here and there a stout parasite strangling the life of a forest tree. But though it is admitted that Nature has played too conspicuous a part in hospital development, and purposed skill has been too often at a discount, yet all this is capable of reformation, and may easily be reformed, provided that rightly chosen persons set about the work in a right way.

Perhaps it is just here that difficulties will begin. Everybody is under the impression that the Charity Organization Society is the parent of the proposed Hospital Committee. Now, nobody loves the parent, and it may not be very easy, therefore, to induce in the proper persons feelings of affection and confidence towards the child. But without such feelings of confidence and esteem it is quite impossible that any good work can be done. The Lords' Committee will have no power to summon witnesses or to compel their attendance. Even if it could compel the attendance of unwilling witnesses, it has not the means of employing skilful cross-examiners to extract the truth from them. Unless, therefore, such steps are taken as will secure the confidence and willing help of hospital managers and officials, the only result of the Committee's inquiries will be a waste of time and public money. It would have been very much better, indeed, if the hospitals themselves had initiated a movement for investigation. The reasons for the Hospitals Association delaying action in this matter are well known. As representing hospital managers it knows how much is being done in various ways to effect reforms from within. Till the various movements in this direction have had time to mature many feel an enquiry by Commission to be of doubtful utility. Hence action has been purposely delayed. As a matter of fact, the hospitals have, through the agency of the Association, devoted much time, labour, and money to those very questions which will be investigated by the Committee. There is no doubt whatever that in sympathy with hospitals and in practical acquaintance with everything that appertains to their management, the Hospitals Association is as superior to the Charity Organization Society as the latter body is to a second-rate detective in ferreting out the delinquencies of a begging letter impostor.

The Committee must on no account allow itself to appear as the tool and creature of the Charity Organization Society. On this point it must set itself right with the public and the hospitals at once. There is before it a work to be done of great and enduring value. If it proves itself equal to the occasion it will place medical charities on such a sound financial basis and in such a favourable light that the public confidence and support will increase immeasurably. But it may entirely fail to rise to the height of its great opportunity; and nothing will so infallibly ensure its failure as its appearance on the public stage in the capacity of a performing bear led by so sinister a master as the Charity Organization Society. We have, of course, no feeling of ill-will against that Society, although we thus speak. There is to be a Lords' Committee, and what we wish for it is that it shall accomplish the work that lies before it with advantage to the public and with honour to itself. But in order to do this it must separate itself entirely from cliques and leaders, and throw itself loyally and with confidence upon the good sense, good faith, and honesty of those who know hospital life and management, and who alone can make its work of great and enduring value.

